# Epilepsy classification using artificial intelligence: A web‐based application

**DOI:** 10.1002/epi4.12800

**Published:** 2023-08-22

**Authors:** Ali A. Asadi‐Pooya, Davood Fattahi, Nahid Abolpour, Reza Boostani, Mohsen Farazdaghi, Mehrdad Sharifi

**Affiliations:** ^1^ Epilepsy Research Center Shiraz University of Medical Sciences Shiraz Iran; ^2^ Department of Neurology, Jefferson Comprehensive Epilepsy Center Thomas Jefferson University Philadelphia Pennsylvania USA; ^3^ Department of Computer Science Engineering and Information Technology Shiraz University Shiraz Iran; ^4^ Vice‐Chancellery for Treatment Affairs Shiraz University of Medical Sciences Shiraz Iran; ^5^ Emergency Medicine Department, School of Medicine Shiraz University of Medical Sciences Shiraz Iran; ^6^ Emergency Medicine Research Center Shiraz University of Medical Sciences Shiraz Iran

**Keywords:** computer, EEG, epilepsy, machine learning, seizure

## Abstract

**Objective:**

The purpose of the current endeavor was to evaluate the feasibility of using easily accessible and applicable clinical information (based on history taking and physical examination) in order to make a reliable differentiation between idiopathic generalized epilepsy (IGE) versus focal epilepsy using machine learning (ML) methods.

**Method**s**:**

The first phase of the study was a retrospective study of a prospectively developed and maintained database. All patients with an electro‐clinical diagnosis of IGE or focal epilepsy, at the outpatient epilepsy clinic at Shiraz University of Medical Sciences, Shiraz, Iran, from 2008 until 2022, were included. The first author selected a set of clinical features. Using the stratified random portioning method, the dataset was divided into the train (70%) and test (30%) subsets. Different types of classifiers were assessed and the final classification was made based on their best results using the stacking method.

**Results:**

A total number of 1445 patients were studied; 964 with focal epilepsy and 481 with IGE. The stacking classifier led to better results than the base classifiers in general. This algorithm has the following characteristics: precision: 0.81, sensitivity: 0.81, and specificity: 0.77.

**Significance:**

We developed a pragmatic algorithm aimed at facilitating epilepsy classification for individuals whose epilepsy begins at age 10 years and older. Also, in order to enable and facilitate future external validation studies by other peers and professionals, the developed and trained ML model was implemented and published via an online web‐based application that is freely available at http://www.epiclass.ir/f‐ige.

**Key points**

- A total number of 1445 patients were studied; 964 with focal epilepsy and 481 with idiopathic generalized epilepsy.
- The stacking classifier led to better results than the base classifiers in general.
- This algorithm has the following characteristics: precision: 0.81, sensitivity: 0.81, and specificity: 0.77.

## 1 INTRODUCTION

The prevalence of epilepsy is about 7 per 1000 people worldwide.^1^ However, epilepsy is not a single diagnostic entity; various brain disorders may cause different types of epileptic seizures and syndromes. In order to competently diagnose and manage “epilepsy,” healthcare professionals must go beyond merely identifying the occurrence or likelihood of recurrent seizures. It is important to make an attempt to diagnose and specify the syndromes and types of epilepsy.^2^ Any healthcare professional, who is dealing with adult patients with epilepsy (PWE), should at least be able to differentiate two general categories of epileptic syndromes/types from one another: idiopathic generalized epilepsies (IGEs) and focal epilepsies.^2^

Making a diagnosis of an epilepsy syndrome or type (eg, IGE vs focal epilepsy) often needs a knowledgeable physician, who is able to obtain a detailed clinical history, access to electroencephalography (EEG), and expertise to interpret an EEG correctly, and access to neuroimaging [brain magnetic resonance imaging (MRI)] and expertise to review it correctly. This constellation of resources is not always available, even in the most developed nations. Misdiagnosis of epilepsy remains common and the consequences for patients are significant (eg, inappropriate treatments, social restrictions, etc.).^3^, ^4^ Importantly, mistakes in the interpretation of EEGs are common and this is an important contributor to the misdiagnosis of epilepsy type.^5^ Therefore, it would be ideal to have an easily accessible and applicable resource to enable a healthcare professional, who is not an expert in the field, but who is dealing with PWE, to make a reliable differentiation between IGE versus focal epilepsy. This syndromic diagnosis establishes the basis for the treating healthcare professional to decide on an appropriate treatment strategy and also to explain the prognosis for patients and their caregivers.^2^

In recent years, artificial intelligence (AI) and machine learning (ML) methods have been broadly utilized in medicine, providing affordable and efficient resources for the diagnosis and treatment of various medical problems.^6^, ^7^ Specifically for epilepsy, many different methods and algorithms (based on AI and ML) have been developed for different purposes.^8^, ^9^ Classification of epilepsy types has been one of the most attractive topics in this field, for which various algorithms have been introduced in recent years. However, most of these methods are based on EEG analysis or neuroimaging data, which may not be available everywhere.^10^, ^11^ On the contrary, clinical information (based on history taking and physical examination) is one of the most informative and valuable data sources for the diagnosis of epilepsy. However, this has been rarely investigated and utilized in ML and AI algorithms.

The purpose of the current endeavor was to evaluate the feasibility of using easily accessible and applicable clinical information (based on history taking and physical examination) in order to make a reliable differentiation between IGE versus focal epilepsy using ML methods. We developed a pragmatic algorithm aimed at facilitating epilepsy classification for individuals whose epilepsy begins at age 10 years and older. Earlier‐onset epilepsy was excluded as some childhood syndromes are complex with regard to diagnosis and would not readily fit into a broader diagnostic and therapeutic scheme designed for adolescents and adults. Also, in order to enable and facilitate external validation studies by other peers and professionals, the developed and trained ML model was implemented and published via an online web‐based application.

## 2 METHODS

### 2.1 Data collection

The first phase of the study was a retrospective study of a prospectively developed and maintained database. All patients with an electro‐clinical diagnosis of IGE or focal epilepsy, at the outpatient epilepsy clinic at Shiraz University of Medical Sciences, Shiraz, Iran, from 2008 until 2022, were included. The first author diagnosed the patients based on clinical features (history and physical examination), EEG, and MRI findings.

Age at seizure onset, sex, seizure semiology, a history of febrile convulsion in childhood, a family history of epilepsy, a history of major head injury (eg, with loss of consciousness for more than 24 hours or with intracranial hemorrhage or with depressed skull fracture‐based on the patient's report), other medical comorbidities, physical examination (eg, any obvious focal neurological sign, microcephaly, etc.), and epilepsy syndrome were registered in the database for all patients.

### 2.2 Clinical features

The first author selected a set of clinical features that are (1) easily obtainable even by people who are not experts in the field and (2) yet are helpful in making a diagnosis of epilepsy type/syndrome (differentiating focal epilepsy from IGE) based on the previous literature. While there are some other clinical features [eg, an exact diagnosis of seizure types (eg, focal seizure with impaired awareness vs absence seizures)] that are very helpful in differentiating focal epilepsy from IGE, these need a skillful and knowledgeable expert; therefore, we did not include these features. Similarly, we did not include EEG and imaging findings for the very same reason.

The selected features included:

- Age at seizure onset: IGE usually begins in childhood/adolescence/young adulthood. Focal epilepsy may begin at any age. Seizures in IGE rarely begin after 25 years of age.^12^, ^13^
- Sex: Female patients often outnumber males in IGEs. The sex (female to male) ratio of the whole cohort of patients with IGE is about 1.5 in various studies.^14^, ^15^, ^16^ On the contrary, men may have a greater predisposition to behaviors that cause brain injuries and acquired (focal) epilepsy. Furthermore, some of the structural focal epilepsies may be more frequent in men (eg, focal cortical dysplasia, perinodular heterotopia).^17^
- A history of febrile convulsion: Several studies have shown a potential relationship between a history of febrile convulsion in early childhood and focal epilepsy (ie, temporal lobe epilepsy with mesial temporal sclerosis) later in life.^18^
- A family history of epilepsy: IGEs have genetic underpinnings while many focal epilepsies are acquired in nature (and some are genetic).
- A history of major head injury: Traumatic brain injury has been recognized as a cause of epilepsy since antiquity, and it remains one of the most common and important causes of acquired (focal) epilepsy today.^19^
- Medical comorbidity: Medical problems (eg, cerebrovascular disorders, cancers, autoimmune disorders, etc.) may cause focal epilepsy.
- Aura: Aura type may help differentiate between IGE and focal epilepsy.^20^ In our database, we have classified and coded aura as follows: “No aura”, “Indescribable feeling”, “Dizziness”, “Fear/Nervousness/Anxiety/Adrenaline rush”, “Cognitive/Deja vu/Jamais vu/Forced thought”, “Epigastric/Abdominal/Nausea”, “Elementary visual”, “Complex visual”, “Elementary auditory”, “Complex auditory”, “Olfactory”, “Gustatory / Taste”, “Left focal sensory”, “Right focal sensory”, “Other sensory”, “Headache”, and “Other”.
- Tongue biting: While ictal injury could be seen in both IGE and focal epilepsies, tongue injury was more frequently reported by patients with temporal lobe epilepsy compared with that by patients with IGE.^21^
- Physical and neurological examination: An idiopathic epilepsy syndrome (eg, IGE) has no underlying structural brain lesion or other neurological signs and symptoms (by definition),^22^ but focal epilepsies may be associated with other abnormalities in examination (eg, focal neurological deficits).

### 2.3 Data preparation

Using the stratified random portioning method, the dataset was divided into the train (70%) and test (30%) subsets. Before training the classifiers, a simple constant imputer addressed the occasional missing values, and the data were standardized using a robust scaler. This scaler offsets the median and scales the data based on the interquartile range. Centering and scaling were applied independently on each feature by computing the required statistics on the samples in the training set.

### 2.4 Classification method

Different types of classifiers were assessed and the final classification was made based on the stacking method. In other words, the proposed classification approach benefited from multiple well‐known classifiers, and their results were given to a stacking classifier as an ensemble method to perform the final classification using the best results. The Stacking classifier (also known as stacked generalization) utilizes a combiner model to aggregate the prediction of multiple other learning algorithms (also called base estimators). The base estimators are trained independently on the data, and subsequently, their outcomes are utilized to train the combiner model to produce a final prediction. The stacking method typically outperforms any single one of the base models.^23^

In the present study, two types of base estimators were involved: three classic classifiers including Support Vector Machine (SVM), Logistic Regression (LogReg), and K‐Nearest Neighbors (KNN), and five Decision Tree‐based ensemble classifiers including Random Forest (RanFor), Gradient Boosting (GradBoost), Adaptive Boosting (AdaBoost), Bagging, and Extremely Randomized Trees (ExtRa Trees). Hyperparameters of the initial classifiers and the final Stacking classifier were trained using grid search and the best ones on the five‐fold cross‐validation are reported in Table S1.

### 2.5 Implementation

All the algorithms were implemented in Python 3.9 using the Scikit‐Learn package. The operating system was Microsoft Windows 10 × 64, on hardware with Intel (R) Core (TM) i5‐8250U CPU @ 1.60 GHz, 1800 MHz, 4 Cores, 8 logical processors, and 8.00 GB of installed physical memory (RAM). An online application was developed. The front‐end user interface (UI) is a simple HTML code that receives clinical inputs from users. Then, using the Flask module, the inputs are given to the back‐end Python code (dealing with the trained model), and the final prediction is returned back to be shown on the UI. The application is accessible via the following link: http://www.epiclass.ir/f‐ige.

## 3 RESULTS

A total number of 1445 patients were studied; 964 with focal epilepsy and 481 with IGE. Data S1 is the full dataset of patients. Table 1 shows the clinical characteristics of the patients. Eight of the selected variables and features were significantly different between the two groups (IGE vs focal epilepsy) in our database. The history of febrile seizure in childhood was not significantly different between the two groups, but we included this feature in the algorithm based on the previous literature.

**TABLE 1:** Clinical characteristics of the patients.

Feature	Focal epilepsy (N = 964)	IGE (N = 481)	*P*‐value
Age at seizure onset, years (*t*‐test)	23.9 ± 13.3	15.8 ± 4.9	**0.0001**
Aura (Pearson Chi‐square)	489 (50.7%)	62 (12.9%)	**0.0001**
Sex (female:male) (Fisher's exact test)	415: 549	305: 176	**0.0001**
History of febrile convulsion (Fisher's exact test)	61 (6.3%)	36 (7.5%)	0.506
Family history of epilepsy (Fisher's exact test)	198 (20.5%)	205 (42.6%)	**0.0001**
History of major head injury (Fisher's exact test)	125 (12.9%)	7 (1.4%)	**0.0001**
Medical comorbidity (Fisher's exact test)	224 (23.2%)	68 (14.1%)	**0.0001**
Abnormal physical examination (Fisher's exact test)	76 (7.9%)	5 (1.0%)	**0.0001**
History of tongue biting with seizures (Pearson Chi‐square)	199 (20.6%)	118 (24.5%)	**0.007**

*Note*: Significant *P*‐values are in bold.

Abbreviation: IGE, idiopathic generalized epilepsy.

The classification results are summarized in Table 2. The stacking classification led to better results than the base classifiers in general (considering precision, sensitivity, specificity, and F1‐score). The confusion matrix of the stacking classifier is depicted in Figure S1. Generally, the results showed a considerable effectiveness in utilizing the selected clinical information in the classification of epilepsies (focal epilepsy vs IGE).

**TABLE 2:** Summary of the classification results.

Classifiers	Precision			Sensitivity			Specificity			F1‐score		
	FE	IGE	Avg	FE	IGE	Avg	FE	IGE	Avg	FE	IGE	Avg
Stack	0.87	0.71	0.81	0.85	0.74	0.81	0.74	0.85	0.77	0.86	0.72	0.81
SVM	0.83	0.68	0.78	0.85	0.66	0.79	0.66	0.85	0.72	0.84	0.67	0.78
LogReg	0.83	0.67	0.78	0.84	0.66	0.78	0.66	0.84	0.72	0.84	0.67	0.78
KNN	0.87	0.66	0.80	0.76	0.80	0.78	0.76	0.80	0.78	0.84	0.71	0.79
RanFor	0.85	0.69	0.80	0.84	0.70	0.79	0.70	0.84	0.77	0.85	0.69	0.80
GradBoost	0.88	0.68	0.80	0.82	0.78	0.80	0.75	0.84	0.77	0.86	0.71	0.80
AdaBoost	0.87	0.69	0.80	0.83	0.78	0.80	0.75	0.84	0.77	0.86	0.72	0.81
Bagging	0.86	0.68	0.80	0.83	0.72	0.79	0.72	0.83	0.76	0.84	0.70	0.79
ExtRa Trees	0.82	0.71	0.78	0.89	0.60	0.79	0.60	0.89	0.70	0.85	0.66	0.79

*Note*: Each row represents a classifier while their precision, sensitivity, specificity, and F1‐score are in the columns for focal epilepsy (FE), idiopathic generalized epilepsy (IGE), and their average.

## 4 DISCUSSION

In the current endeavor, we developed a pragmatic algorithm aimed at facilitating epilepsy classification (IGE vs focal epilepsy) for individuals whose epilepsy begins at age 10 years and older (http://www.epiclass.ir/f‐ige). This algorithm has the following characteristics: precision: 0.81, sensitivity: 0.81, and specificity: 0.77. The most important feature of this algorithm is that it could be used by people who are not experts in epilepsy diagnosis (eg, family physicians, internists, etc.) but may deal with PWE.

In one study of 350 adult PWE (mostly treated by neurologists, presumably after ordering EEG and brain imaging studies), 29% were taking wrong antiseizure medications (misclassified epilepsy type).^4^ In another study of 324 patients, the overall misdiagnosis rate was 26%, with incomplete history taking and misinterpretation of the EEG equally responsible.^24^ In another study of 200 patients with juvenile myoclonic epilepsy (JME), 49 (24.5%) were misdiagnosed at the first medical evaluation. The physician was a neurologist in 87.8% of cases with misdiagnoses.^25^ Therefore, 0.81 precision by the algorithm using only simple features in the clinical history and physical examination seems very promising in advancing care of PWE by assisting the healthcare professionals in making a correct diagnosis of the epilepsy type.

In our algorithm, the classification results for focal epilepsy were better than those for IGE for all the evaluation parameters (Table 2). This may be caused by the imbalanced number of the samples in the classes. Larger studies including more such clinical features (that are easily recognizable by professionals who are not experts in neurology/epileptology) may provide different and more reliable results and a multicenter study is very much needed to advance this important task. Such a study on individualized prediction of drug resistance and seizure recurrence after medication withdrawal in people with juvenile myoclonic epilepsy was published recently.^26^

While there are other applications to assist healthcare professionals in making a diagnosis of epilepsy type (eg, https://www.epipick.org),^27^ our current algorithm and application have two advantages: first, it is based on ML algorithms on a large dataset of patients; second, it uses only simple and easily accessible clinical features as described before. However, our algorithm and application should be validated externally to show its generalizability; the Epipick application has been validated in multiple studies.^28^, ^29^ The application of AI and ML in medicine has helped healthcare professionals improve the quality of care that they can deliver and has the promise to improve it even more in the near future and beyond.^29^ Of course, AI and ML will not and cannot put healthcare professionals out of business; rather, they will make it possible for such professionals to do their jobs more accurately and leave some time for the human–human interactions that make medicine the rewarding profession we all value.^30^

Our study has some limitations. This was a single‐center study. A single expert (the first author) diagnosed the patients based on clinical features (history and physical examination), EEG, and MRI findings. It would have been more reliable if diagnoses were based on consensus from several experts and follow‐up information including therapeutic responses. Furthermore, the database was unbalanced (964 with focal epilepsy vs 481 with IGE), with a possible impact on the reliability of the training and testing stages of the algorithms.

## 5 CONCLUSION

**FIGURE 1:**
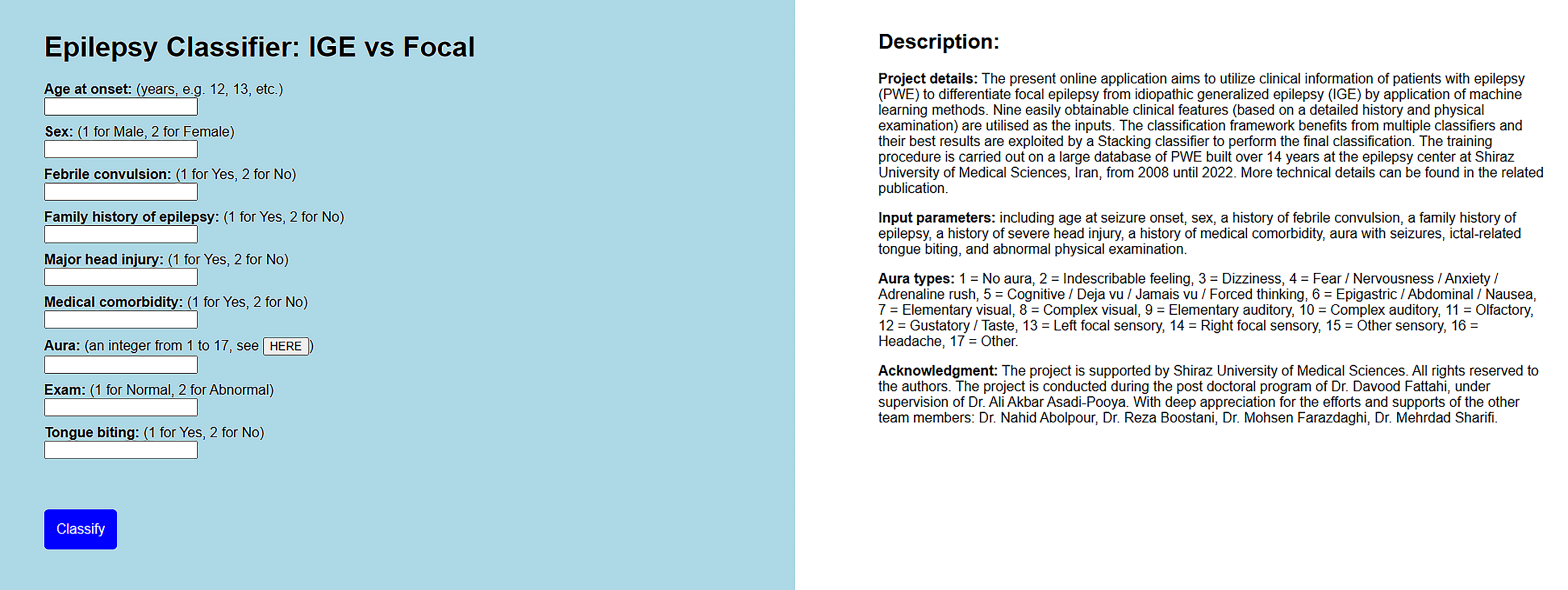
Epilepsy classification using artificial intelligence: a web‐based application.

We developed a pragmatic algorithm aimed at facilitating epilepsy classification for individuals whose epilepsy begins at age 10 years and older. Also, in order to enable and facilitate future external validation studies by other peers and professionals, the developed and trained ML model was implemented and published via an online web‐based application that is freely available at http://www.epiclass.ir/f‐ige (Figure 1). The acceptable classification rate of the proposed framework can promise the feasibility of using clinical features in an affordable and available AI and ML setting to diagnose and classify epilepsy types.

## AUTHOR CONTRIBUTIONS

Ali A. Asadi‐Pooya, M.D. involved in study design, data collection, and manuscript preparation. Davood Fattahi, Ph.D. involved in machine learning process, and manuscript preparation. Other authors collected the data and prepared the manuscript.

## FUNDING INFORMATION

This work was supported by Shiraz University of Medical Sciences. The funding source had no involvement in the study design; in the collection, analysis, and interpretation of data; in the writing of the report; and in the decision to submit the article for publication.

## CONFLICT OF INTEREST STATEMENT

Ali A. Asadi‐Pooya: Honoraria from Cobel Daruo, Actoverco; Royalty: Oxford University Press (Book publication); Grant from the National Institute for Medical Research Development. Others: no conflict of interest.

## ETHICS STATEMENT

This study was conducted with the approval of Institutional Ethics Review Boards at Shiraz University of Medical Sciences (Post‐Doc project by Davood Fattahi, Ph.D. and under the supervision of Ali A. Asadi‐Pooya, M.D.).

## INFORMED CONSENT

The participants gave their informed consent for the use of their data for research purposes.

## Supporting information


Table S1.
Figure S1.

Data S1.

## Data Availability

The data are shared in Data S1.
