# Supplementary material for: Epilepsy classification using artificial intelligence: A web‐based application
Source: Epilepsia Open. 2023 Aug 22;8(4):1362–8. doi: 10.1002/epi4.12800 (PMC10690646; doi:10.1002/epi4.12800)
Supplement: Supplementary file 1 — Table S1. Figure S1. [file EPI4-8-1362-s002.docx]

| **Supplementary Table 1.** Optimized hyperparameters and setting of the base and final classifiers. | |
| --- | --- |
| **Classifier** | **Parameters and settings.** |
| SVM | Regularization parameter: 3.5, kernel coefficient (gamma): scale, shrinking: true, kernel: RBF, decision function: one-vs-rest. |
| LogReg | Inverse of regularization strength: 0.5, penalty: L2, solver: Newton conjugate gradient. |
| KNN | Algorithm: best of (BallTree – KDTree - Brute-force search), Leaf size passed to BallTree or KDTree: 10, number of neighbors: 23, weights: Euclidean distance, |
| RanFor | Criterion: Gini, minimum samples at leaf nodes: 2, minimum samples to split: 8, number of estimators: 20, maximum features at each split: square root of the number of features, bootstrap: true. |
| GradBoost | Criterion: Friedman mean squared error, loss: exponential, number of estimators: 40, learning rate: 0.1. |
| AdaBoost | Algorithm: SAMME.R, learning rate: 1.0, number of estimators: 50. |
| Bagging | Maximum features for each estimator: 95% of features, maximum samples for each estimator: 25% of samples, number of estimators: 50. |
| ExtRa Trees | Criterion: Gini, minimum samples at leaf nodes: 2, minimum samples to split: 6, number of estimators: 20, maximum features at each split: square root of the number of features, bootstrap: false. |
| Stack | Stack method: predict probability, Final estimator: logistic regression. |

| 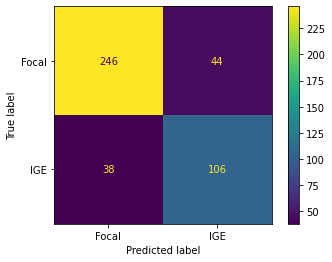 |
| --- |
| **Supplementary Figure 1.** Confusion matrix of the number of true labels versus predicted labels for the Stack classifier’s test evaluation. Idiopathic generalized epilepsy (IGE) and Focal epilepsy are the classes.  **Supplementary Figure 1 legend.** This matrix helps us to find out how many cases are predicted correctly by our classifier. As you can see, 246 focal epilepsy patients were predicted with the correct label, and 44 with the wrong label (predicted as IGE). Also, 106 IGE patients were correctly predicted and 38 were falsely predicted (as focal epilepsy). |
